# Species-specific alternative splicing of *SP110* drives tuberculosis susceptibility in cattle

**DOI:** 10.1186/s13567-025-01644-3

**Published:** 2025-12-12

**Authors:** Ajiao Fan, Ying Guo, Zhunan Li, Xiangchen Dong, Zihan Zhang, Xinyu Wang, Yanliang Song, Jing Han, Jing Yang, Haoxin Wang, Xinyan Zhang, Yong Zhang, Yuanpeng Gao

**Affiliations:** 1 Hainan Institute of Northwest A&F University, Sanya, 572025 Hainan China; 2 College of Veterinary Medicine, Northwest A&F University, Yangling, 712100 Shaanxi China; 3 Key Laboratory of Livestock Biology, Northwest A&F University, Yangling, 712100 Shaanxi China; 4 National Center of Technology Innovation for Dairy, Hohhot, China

**Keywords:** Dairy cows, *Mycobacterium tuberculosis*, *SP110*, alternative splicing

## Abstract

Bovine tuberculosis (bTB) is a chronic debilitating zoonotic disease caused by *Mycobacterium bovis* (*M. bovis*). *M. bovis* exhibits a broad host range, but dairy cattle are particularly susceptible. The *SP110* (speckled protein 110) gene has been demonstrated to be associated with host susceptibility to TB, but whether the susceptibility of dairy cattle to *M. bovis* is correlated with the bovine *SP110* (b*SP110*) gene has not been investigated. Here, we demonstrated that the heightened susceptibility of dairy cattle to bTB was driven by species-specific alternative splicing of the bovine *SP110* (b*SP110*) gene, which resulted in predominant expression of a truncated b*SP110c* variant lacking the SAND domain required for anti-bTB function. Compared to the other two splice variants of b*SP110* gene, b*SP110a* and b*SP110b*, b*SP110c* showing significantly reduced resistance to *M. bovis*. Mechanistically, the ruminant-specific absence of a upstream of the SAND exon (designated as the pre-SAND exon) affects alternative splicing of the b*SP110* gene, consequently promoting b*SP110c* production, while substituting this region with human/equine sequences introduces suppress b*SP110c* and increase b*SP110a/b* expression. Our study provides a new perspective on the genetic basis of the susceptibility of dairy cows to bTB and identifies a potential gene editing region for the breeding of tuberculosis-resistant dairy cows.

**Supplementary Information:**

The online version contains supplementary material available at 10.1186/s13567-025-01644-3.

## Introduction

Bovine tuberculosis (bTB) is a chronic debilitating zoonotic disease caused by *Mycobacterium bovis* (*M. bovis*) [1]. Worldwide, approximately 50 million cattle are infected with bTB annually, resulting in $3 billion in economic losses [2], with 10–15% of human tuberculosis cases attributable to *M. bovis* [3–5]. *M. bovis* is primarily transmitted through inhalation of respiratory aerosols and has the broadest host range within the *Mycobacterium tuberculosis* complex (MTBC), infecting cattle, humans, horses, and other species [6, 7]. However, dairy cattle show heightened susceptibility to *M. bovis* infection compared with other hosts [8–10]. Studies suggest that bTB susceptibility in dairy cattle is associated with specific genomic regions [11]. Identifying genes responsible for susceptibility to bTB in dairy cattle could enhance our understanding of the genetic mechanisms underlying the disease and potentially lead to breeding bTB-resistant dairy cattle [12].

The *SP110* (speckled protein 110) gene has been shown to be associated with host susceptibility to TB [13–15]. Research indicates that h*SP110B*, a splice variant of the human *SP110* gene, can interact with NF-κB to resist *M. tuberculosis* (MTB) infection [16]. The *Ipr1* gene, located on mouse chromosome 1 in the super-susceptibility to TB 1 (sst1) locus, is the mouse homolog of *SP110* [17, 18]. Site-specific knock-in of the murine *Ipr1/SP110* gene mediated by the transcription activator-like effector (TALE) nickase can produce TB-resistant cattle. In vitro and in vivo challenge experiments proved that the transgenic cattle were able to inhibit the growth and multiplication of *M. bovis* [19]*.* These studies demonstrated that the *SP110* gene plays an important role in TB resistance. However, the *SP110* gene exhibits characteristic species-specific structural differences, and can generate splice variants with substantial functional divergence through alternative splicing mechanisms [20–22], potentially influencing the host's susceptibility to TB. The relationship between dairy cattle susceptibility to bTB and the b*SP110* gene structure and splice variants in cattle remains to be determined.

To investigate the relationship between the b*SP110* gene and dairy cattle susceptibility to bTB, we first characterized the splice variants of b*SP110* and their anti-bTB functions. Our study identified three transcript variants of the b*SP110* gene: b*SP110a*, b*SP110b*, and b*SP110c*. B*SP110c* has not been reported in other species, and it confers the weakest bTB resistance compared with b*SP110a* and b*SP110b*. The specific structure of the b*SP110* gene results in the generation of the b*SP110c* splicing variant. Substitution of human or equine sequences upstream of the SAND exon (pre-SAND exon) in the corresponding position of the b*SP110* gene reduced the expression of b*SP110c* and improved the resistance to *M. bovis* in cattle. Our study revealed the genetic factors of bovine susceptibility to *M. bovis* and provided a gene editing site for breeding bTB-resistant cows.

## Materials and methods

### Ethics statement

All animal experiments in this study were approved by the Animal Ethics Committee of Northwest A&F University (Approval No. 2021042) and strictly adhered to the “Guidelines for Animal Care and Use for Research Purposes”. The Holstein cattle blood plasma and healthy 3–6-month-old Holstein fetuses were sourced from Yangling Keyuan Co. Every effort was made to minimize animal pain, suffering, and distress, and reduce the number of animals used.

### Cell culture and transfection

Hela, HEK293T, RAW264.7, and NIH3T3 cells were cultured in high-glucose DMEM supplemented with 10% fetal bovine serum (FBS), while THP-1 cells were maintained in RPMI 1640 with 10% FBS. Bovine fetal fibroblasts (BFFs) were isolated from 3- to 6 month-old Holstein fetuses (Yangling Keyuan Co.). Tissue from the back of the fetus was removed, minced with scalpels under aseptic conditions, plated on 60-mm Petri dishes (Corning Costar), and cultured in DMEM/F12 with 10% FBS. Peripheral blood mononuclear cells (PBMCs) were isolated from bovine plasma (Yangling Keyuan Co.) and induced to differentiate into bovine monocyte-derived macrophages (bMDMs) using granulocyte–macrophage colony-stimulating factor (GM-CSF). BMDMs were cultured in DMEM/F12 with 10% FBS. Plasmid transfections were performed using Lipofectamine 3000 (Invitrogen) according to the manufacturer's protocol.

### 3′RACE-PCR (rapid amplification of cDNA ends)

3′RACE primers were designed according to the predicted mRNA sequence of b*SP110* in the NCBI database. Total RNA was extracted from bMDMs, reverse transcribed according to the instructions of the SMARTer cDNA amplification kit, followed by 3′RACE-PCR to amplify b*SP110* splice variants. The primers used in 3′RACE are shown in Table 1.

**Table 1:** **Primer sequences of 3′RACE PCR amplification**

Primer name	Primer sequence
SP110-3′RACE-NGSP-1	GTCTCCCCTGCCGACAACCCCTAC
SP110-3′RACE-NGSP-2	AAGGAAGGCTCAGTCCAGTGTCCAG
SP110-3′RACE-NGSP-3	CCTGAACCAAGCGACCCAAAGGA
SP110-3′RACE-NGSP-4	ATGATGTCCCTGAACCAAGCGACCCA

### Plasmid construction

Plasmids expressing b*SP110* splice variants were constructed by cloning PCR-amplified b*SP110a*, b*SP110b*, and b*SP110c* fragments, which were obtained using primers based on the b*SP110* splice variant sequences obtained by 3′RACE, into the pCMV-HA vector. Plasmids expressing IRF family members, STING, TBK1, hnRNPA2/B1, and hnRNPF CDS were constructed by amplifying the target sequences from bovine PBMCs, purifying the PCR products, and cloning them into the pCMV-Flag-N vector. Plasmids expressing SRSF1 and SRSF5 were constructed by PCR amplification from Hela cells followed by cloning into the pCMV-Flag-N vector. Plasmids containing the promoter region of the b*SP110* gene were constructed by PCR amplification from bMDMs genomic DNA and subsequent cloning into the pGL4.10 luciferase reporter vector. The bSP110 minigene eukaryotic expression vector, containing exons 1–11 (without introns), partial intron 11, exons 12–14 (with introns), partial intron 14, exons 15–19 (without introns), and 3′UTR sequences, was cloned from the bMDMs genomic DNA into pCMV-Flag-N. To construct chimeric b*SP110* mini-gene vectors with human or equine pre-SAND sequence substitutions, the human pre-SAND exon and its adjacent 5′ intronic sequence were PCR-amplified from Hela cell genomic DNA, while the equine pre-SAND exon and flanking 5′ intron were synthesized by Tsingke Biotechnology Co, Ltd. These isolated human or equine sequences were then used to replace the homologous region in the b*SP110* mini-gene through homologous recombination. The primers used in plasmid construction are shown in Additional file 1 and Additional file 2.

### Electrophoretic mobility shift assay (EMSA)

ISRE upstream and downstream sequences (20 bp) were selected as EMSA probes and the point-mutated ISRE probe sequences were synthesized, and labeled with biotin according to the instructions provided by Beyotime Co. The probes were incubated with the target proteins and EMSAs were performed using a chemiluminescence detection kit according to the instructions. The sequences of EMSA probes are shown in Table 2.

**Table 2:** **Sequences of EMSA probes**

Probe name	Probe sequence (5′-3′)
SP110-ISRE-F	TCCGCAGGATCGGCCCGAGTACTTTCACTTTCACTTTCCTGGAAGCCAGGCCC
SP110-ISRE-R	GGGCCTGGCTTCCAGGAAAGTGAAAGTGAAAGTACTCGGGCCGATCCTGCGGA
SP110-ISREmut-Biotin-F	TCCGCAGGATCGGCCCGAGTACCCTCATCTTCCTTTCCTGGAAGCCAGGCCC-Biotin
SP110-ISREmut-Biotin-R	GGGCCTGGCTTCCAGGAAAGGAAGATGAGGGTACTCGGGCCGATCCTGCGGA-Biotin
SP110-ISRE-Biotin-F	TCCGCAGGATCGGCCCGAGTACTTTCACTTTCACTTTCCTGGAAGCCAGGCCC-Biotin
SP110-ISRE-Biotin-R	GGGCCTGGCTTCCAGGAAAGTGAAAGTGAAAGTACTCGGGCCGATCCTGCGGA-Biotin

### CHIP-qPCR (chromatin immunoprecipitation quantitative PCR)

ChIP assays were performed on human THP-1 cells after transfection with pCMV-Flag-N and pCMV-Flag-IRF3. ChIP DNA was detected by qPCR using primers from the promoter region of hSP110. The assays were performed according to the SimpleChIP^®^ enzymatic chromatin IP kit (CST) instructions. The primer sequences of ChIP-qPCR are shown in Table 3.

**Table 3:** **Primer sequences of ChIP-qPCR**

Primer name	Primer sequence
hSP110-CHIP-qpcr-F	ACTTTCACTTTTCTTTTCTCGGAAG
hSP110-CHIP-qpcr-R	GGGACAGGGATCACTCCTCAAGATT

### MTB culture and infection

*M. tuberculosis* strain MTB H37Ra was purchased from the American Type Culture Collection (ATCC, 25177), and *M. bovis* was obtained from the Guo Aizhen group at Huazhong Agricultural University. H37Ra was maintained in 7H9 medium, while *M. bovis* was cultured in 7H11 medium. Both media were supplemented with 10% oleic albumin dextrose catalase (Solarbio) and 0.5% glycerol. The required number of bacteria was pelleted by centrifugation at 4500 × *g* for 5 min, washed with phosphate-buffered saline (PBS), and resuspended in PBS for subsequent cell infection. All cells were infected at a multiplicity of infection (MOI) of 10. Post-infection, cells were incubated at 37 °C with 5% CO₂ for 4 h, washed three times with PBS to remove non-internalized mycobacteria, and 50 μg/mL of gentamicin was added to the culture medium for 1 h. Afterwards, the culture medium was replaced with fresh and the cell culture was continued for the specified time.

### CFU assay for MTB

RAW264.7 cells were infected with M. bovis at an MOI of 10 for 24 h or 36 h, followed by washing three to five times in PBS and intracellular bacteria were released by cell lysis in PBS containing 0.25% Triton X-100 for 10 min at room temperature. The lysates were serially diluted in PBS and plated in triplicate on 7H11 agar plates. Agar plates were incubated at 37 °C for three weeks and colonies were counted.

### Luciferase reporter assay

HeLa cells cultured in 24-well plates were co-transfected with the luciferase reporter vector (90 ng) and pRL-TK control vector (10 ng) using Lipofectamine 3000 (Invitrogen). At 24 h post-transfection, cells were harvested and lysed. The Dual-Luciferase Reporter Assay System (Promega) was employed to sequentially measure firefly and Renilla luciferase activities, with their ratio representing promoter activity. Three independent biological replicates were performed, each with technical duplicates.

### Real-time quantitative PCR (RT-qPCR)

Total RNA was isolated using RNAiso Plus (Takara) and reverse-transcribed into cDNA using HiScript III RT SuperMix for qPCR according to the manufacturer’s instructions (Vazyme). ChamQ SYBR qPCR master mix (Vazyme) was used for quantitative real-time PCR analysis. The relative mRNA expression of target genes were normalized to GAPDH and calculated using the 2^−△△Ct^ method. The primers used in RT-qPCR are shown in Table 4.

**Table 4:** **Primer sequences of qPCR amplification**

Primer name	Primer sequence (5′-3′)
qPCR-Va-F	CATTTTACAAGGCTTCCGACTTTGG
qPCR-Va-R	TTATATGGATCCTGAAGTTGGGATG
qPCR-Vb-F	GCTGCTGCAGAACGGAATTTTGTTC
qPCR-Vb-R	CACATGGTAACTCTGCAACGTGGTG
qPCR-Vc-F	CCGGGAAAAAGCGAGCAGCCTCATC
qPCR-Vc-R	TCACTTTAGATCTACATCACCTGTG

### Cytokine ELISAs

RAW264.7 cells were infected with *M. bovis* at an MOI of 10 for 36 h. The culture supernatants were then collected, filtered through a 0.22 μm filter, and analyzed for mouse IL-1β, TNF-α, IL-10, and IL-6 levels using commercial ELISA kits (Proteintech) following the manufacturer's instructions.

### Statistical analysis

The trials were conducted three times, and data are presented as the mean ± SD. Statistical significance was determined using Student’s *t* test, and *P* < 0.05 was deemed to be statistically significant.

## Results

### MTB infection activates b*SP110* transcription via the cGAS- STING- IRF3 signaling pathway

**Figure 1:**
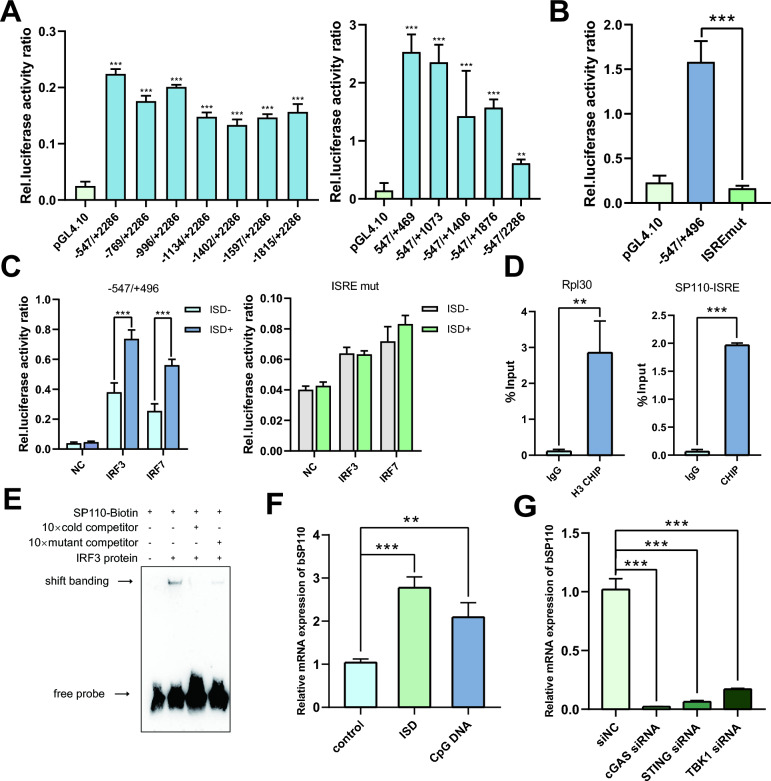
**MTB infection activates b*SP110*** **transcription via the cGAS/STING/IRF3 pathway.** **A** Activity of the truncated promoter, focusing on sequences upstream and downstream of the transcription start site (TSS). **B** The ISRE plays a key role in ISD induction and regulation of b*SP110* transcription. **C** Effect of IRF3 and IRF7 on ISD induction and regulation of b*SP110* transcription. **D** Enrichment of IRF3 in the promoter region of *SP110* was detected by ChIP-qPCR enrichment of the *Rpl30* gene of H3 relative to IgG. **E** IRF3 specifically binds to the *SP110* promoter as detected by EMSA. **F** ISD and CpG DNA can effectively activate b*SP110* transcription. **G** Effect of RNAi of cGAS/STING/TBK1 pathway protein on transcription of b*SP110*. Data were analyzed by *t*-test and presented as the mean ± SD of three independent experiments (*P < 0.05, **P < 0.01, ***P < 0.001).

Earlier studies showed that the transcript abundance of the b*SP110* gene increased rapidly within 2–6 h after infection of bovine monocyte-derived macrophages (bMDMs) with *M. bovis* [23]. To identify the signaling pathways that activate b*SP110* transcription in response to *M. bovis* infection, we cloned and characterized the b*SP110* core promoter. The dual-luciferase reporter assay results demonstrated that the −547/ + 469 region of the bSP110 promoter exhibited the highest transcriptional activity, defining it as the core promoter of the b*SP110 *gene (Figure 1A). JASPAR database prediction revealed an interferon-stimulated response element (ISRE) within the b*SP110* core promoter region. Mutation of this motif significantly impaired promoter-driven gene expression (Figure 1B).

To identify transcription factors binding to the ISRE, we performed JASPAR database and UCSC database association analyses and identified IRF3 and IRF7 as potential transcription factors with the ability to bind to the ISRE, with IRF3 being the most likely (Figure 1C). Electrophoretic mobility shift assay (EMSA) and chromatin immunoprecipitation-quantitative real time PCR (CHIP-qPCR) experiments showed that IRF3 bound to the ISRE in the promoter of b*SP110* (Figures 1D, E), supporting the role of IRF3 in b*SP110* transcription. The qPCR results showed that interferon-stimulated DNA (ISD) and cytosine-phosphate-guanine DNA (CpG DNA) of mycobacterial origin were able to activate b*SP110* transcription (Figure 1F), which suggested that *M. bovis* infection might induce b*SP110* transcription through the cGAS/STING/IRF3 intracellular DNA recognition pathway. To test this hypothesis, we performed RNA interference (RNAi) assays in bMDMs on STING and TANK-binding kinase-1 (TBK1). The results showed that b*SP110* mRNA levels were significantly reduced by RNAi silencing of cGAS, STING and TBK1 (Figure 1G). In summary, our findings suggest that transcription of b*SP110* can be activated through the cGAS/STING/IRF3 pathway.

### Expression levels of the three splice variants of the bovine *SP110* gene differ after MTB infection

**Figure 2:**
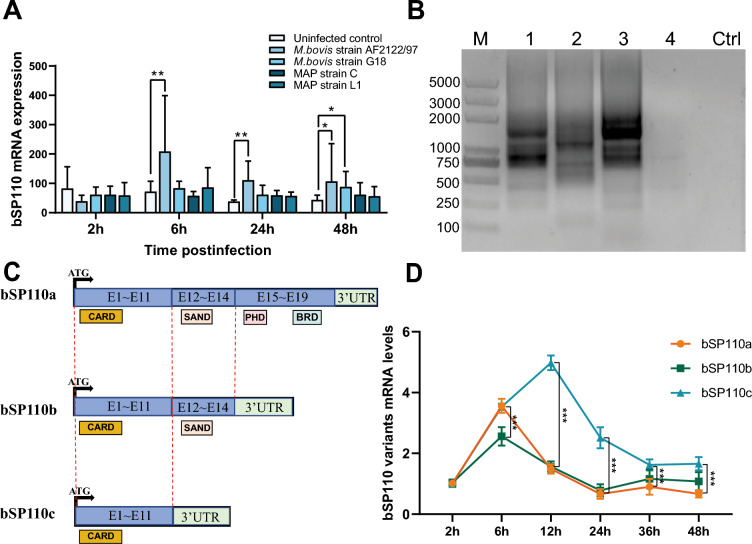
**Expression levels of the three splice variants of the bovine** ***SP110*** **gene after MTB infection.** **A** Detection of b*SP110* mRNA levels in bovine monocyte-derived macrophages infected with *M. bovis* and *M. avium* subspecies paratuberculosis (MAP) GEO accession, GSE104211. **B** PCR results of 3′RACE; M, Trans 2 K Plus (II) DNA Marker; lanes 1–5, PCR results of different primers; Ctrl, control. **C** Schematic of the b*SP110* domain. **D** Detection of b*SP110* mRNA levels in bMDMs infected by H37Ra. Data were analyzed by *t*-test and presented as the mean ± SD of three independent experiments (*P < 0.05, **P < 0.01, ***P < 0.001).

Transcriptome analysis of NCBI GEO Datasets revealed significant activation of b*SP110* transcription by *M. bovis* infection (Figure 2A). To characterize the splice variants generated following b*SP110* transcription, we performed 3′RACE-PCR with sequence analysis of the amplified products and identified three splice variants of b*SP110* designated as b*SP110a*, b*SP110b* and b*SP110c* (Figure 2B). Amino acid sequences of the three variants were predicted for conserved domains using the NCBI Conserved Domain Search tool, and the functional domains of the three variants are shown in Figure 2C. Notably, the C-terminal domains were progressively truncated across the variants, with a deletion of the core functional domain, SAND, from b*SP110c*. Temporal expression profiling in H37Ra-infected bMDMs demonstrated that b*SP110c* had significantly higher mRNA levels than b*SP110a* and b at 12 h and 24 h post-infection (Figure 2D).

### Variants b*SP110a* and b*SP110b* show greater anti-bTB activity than b*SP110c*

**Figure 3:**
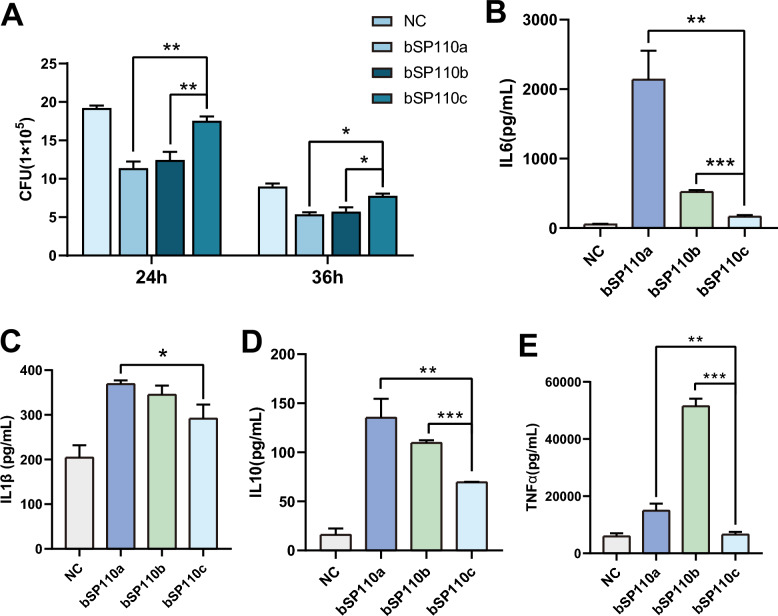
**The variants, b*SP110a* and b*SP110b*, showed greater anti-bTB activity than b*SP110c***. **A** *M. bovis* CFU from RAW264.7 macrophages overexpressing b*SP110a, b, or c* after infection for 24 h and 36 h. **B**–**E** Expression of IL6, IL1β, IL10, and TNFα in RAW264.7 macrophages overexpressing b*SP110a, b and c* after *M. bovis* infection (detected by ELISA). Data were analyzed by *t*-test and presented as the mean ± SD of three independent experiments (*P < 0.05, **P < 0.01, ***P < 0.001).

To explore the bTB-resistance of the three b*SP110* splice variants, we overexpressed b*SP110a*, b*SP110b*, and b*SP110c* in the mouse macrophage cell line, RAW264.7, and infected the cells with *M. bovis*. The CFU results showed reduced intracellular *M. bovis* infection in all three b*SP110* splice variants, but transfection of b*SP110a* and b*SP110b* resulted in a significantly larger decrease in intracellular *M. bovis* compared to b*SP110c* at 24 h and 36 h after infection (Figure 3A). ELISAs demonstrated that all three variants, b*SP110c*, b*SP110a* and b*SP110b*, significantly promoted the expression of IL6, IL1β, IL10, and TNFα in macrophages after *M. bovis* infection (Figures 3B–E).

### The possible presence of ESS in exon 11 of the bovine *SP110* gene could lead to the production of b***SP110c***

**Figure 4:**
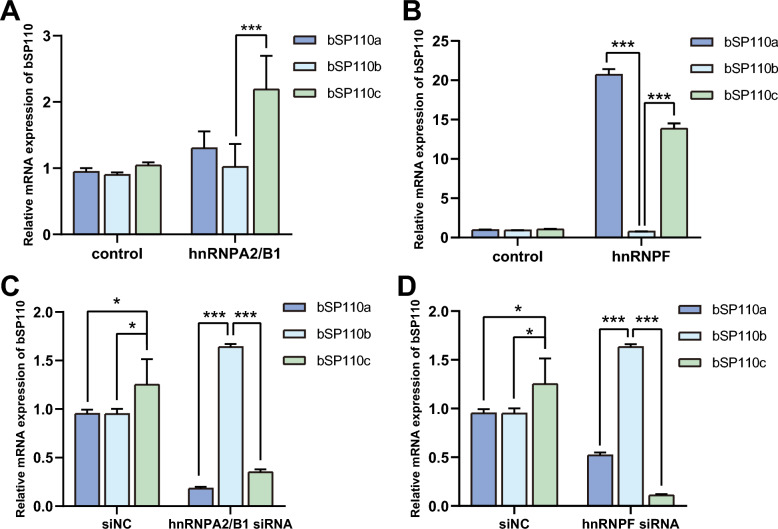
**An ESS was found in exon 11 of the b*SP110*** **gene and resulted in the production of b*SP110c***. **A** Effect of hnRNPA2/B1 overexpression on mRNA levels of the three *SP110* variants. **B** Effect of hnRNPF overexpression on mRNA levels of the *SP110* variants. **C** Effect of hnRNPA2/B1 RNAi on mRNA levels of the *SP110* variants. **D** Effect of hnRNPF RNAi on mRNA levels of the *SP110* variants. Comparisons were analyzed for significant differences by *t*-test and presented as the mean ± SD of three independent experiments (*P < 0.05, **P < 0.01, ***P < 0.001).

Exon splicing silencers (ESS) are often bound by hnRNPs, resulting in exon skipping [24]. To determine whether the production of b*SP110c* was a result of the presence of an ESS, we constructed a b*SP110* minigene vector and overexpressed it with hnRNPA2/B1 and hnRNPF. The results showed that when hnRNPA2/B1 and hnRNPF were overexpressed, b*SP110a* and b*SP110c* mRNAs were increased, while b*SP110b* mRNA was decreased (Figures 4A, B). When hnRNPA2/B1 and hnRNPF expressions were silenced by RNAi, b*SP110a* and b*SP110c* mRNA levels were significantly decreased, while b*SP110b* mRNA was significantly upregulated (Figures 4C, D). The results showed that hnRNPA2/B1 and hnRNPF effectively inhibited the splicing of b*SP110a* and b*SP110c* but promoted the splicing of b*SP110b*, indicating that there may be an ESS binding to the splicing factors hnRNPA2/B1 and hnRNPF in exons 11 and 19 of b*SP110*, resulting in the formation of b*SP110a* and b*SP110c*.

### Substitution of human or equine pre-SAND exon in the corresponding position of the b*SP110* gene reduces the expression of b*SP110*c

**Figure 5:**
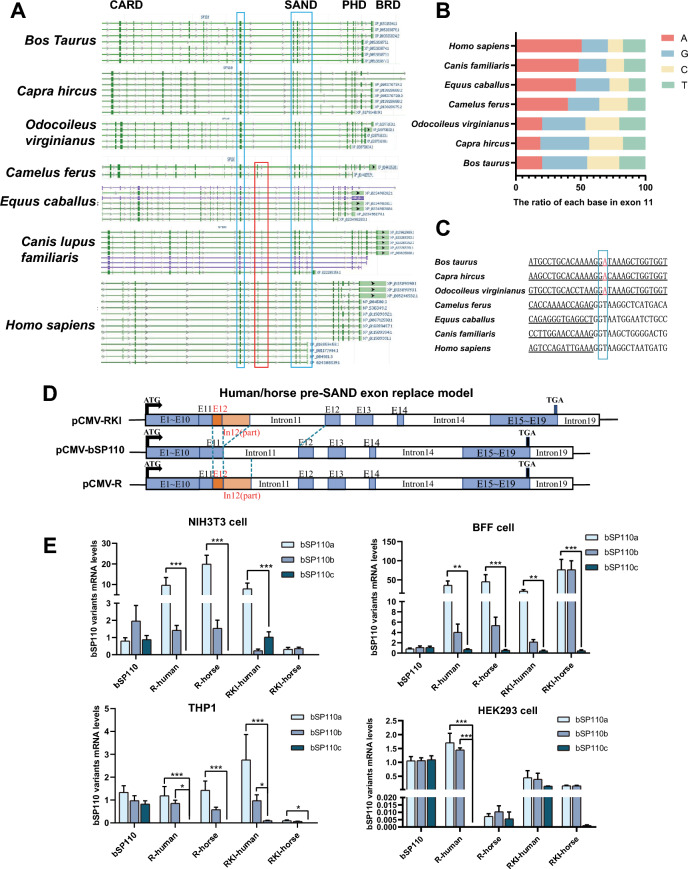
**Replacing the human or horse pre-SAND exon at the corresponding position in the bovine** ***SP110*** **gene reduces b *SP110c*** **expression**. **A** Amino acid sequence alignment of *SP110* from different species. The red box indicates the 5′-terminal pre-SAND exon adjacent to the SAND domain of the *SP110* gene of non-ruminant species. The blue box indicates the homologous exon sequence at both ends of the pre-SAND exon. **B** Base composition comparison of corresponding sequences of the pre-SAND exon of different species. **C** Comparison of the intron splice recognition sites associated with the pre-SAND exon of different species. **D** Diagram showing the structure of the b*SP110* minigene vector and the corresponding mutant vector. **E** Effect of substitution of the human or equine pre-SAND exon on *SP110* splicing in different cell lines. Data were analyzed by *t*-test and presented as the mean ± SD of three independent experiments (*P < 0.05, **P < 0.01, ***P < 0.001).

We compared the DNA sequences upstream and downstream of the SAND domain exon of the *SP110* gene in different species using NCBI and found that, compared with other species, the sequences immediately adjacent to the 5' end of the SAND exon of cows, sheep, and deer were missing (Figure 5A); this region, we designated as the ‘pre-SAND’ exon. The base composition of the pre-SAND exon of non-ruminant species showed typical A-rich features compared with the corresponding sequences of ruminants (Figure 5B). There was a point mutation in the splice recognition site (GU-AG) of the ruminant b*SP110* intron 11 (Figure 5C). These results suggest that the ruminant *SP110* gene has a unique genetic structure. To determine whether the generation of b*SP110c* was related to deletion of the pre-SAND exon, we constructed two vectors in which the corresponding position of the b*SP110* minigene was replaced by the human and equine pre-SAND exon and the partial sequence of intron 12 and named them b*SP110*-Rhuman and b*SP110*-Rhorse. To ensure the retention of the splice element, we also constructed two vectors named b*SP110*-RKIhuman and b*SP110*-RKIhorse (Figure 5D). Quantitative PCR results showed that substitution of the human or equine pre-SAND exon sequence in the bovine *SP110* gene upstream of the SAND exon effectively reduced expression of the b*SP110c* splice variant transcript (Figure 5E).

### An exon splicing enhancer (ESE) in the pre-SAND exon of the human and equine *SP110* genes regulates alternative b*SP110* splicing

**Figure 6:**
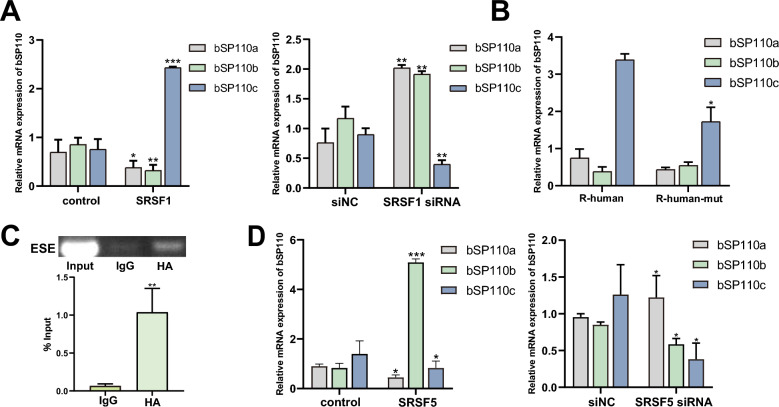
**The ESE in the pre-SAND exons of the human and horse** ***SP110*** **genes can regulate the alternative splicing of the bovine** ***SP110*** **gene.** **A** Effect of SRSF1 overexpression and RNAi on *SP110* variant mRNA levels. **B** ESE site mutation can affect SRSF1 regulation of *SP110* splicing. **C** Results of RNA immunoprecipitation. **D** Effect of SRSF5 overexpression and RNAi on *SP110* variant mRNA levels. Data were analyzed by *t*-test and presented as the mean ± SD of three independent experiments (*P < 0.05, **P < 0.01, ***P < 0.001).

To determine why substitution of the human or horse pre-SAND exon sequence in the b*SP110* gene reduced b*SP110*c generation, we first performed exon splicing enhancer (ESE) analysis of the human and equine *SP110* pre-SAND exon in ESE Finder software. Both human and equine pre-SAND exons were predicted to have ESE elements that can bind to serine/arginine-rich (SR) proteins (Additional file 3), we selected SRSF1 as a possible candidate for binding to the ESE of the pre-SAND exon of human *SP110* and SRSF5 as a possible candidate for binding to the ESE of the equine pre-SAND exon. The results showed that overexpression of SRSF1 effectively promoted the production of b*SP110c* mRNA and reduced the production of b*SP110a* and b*SP110b*. In contrast, when cells were transfected with anti-SRSF1 siRNA, there was a significant decrease in b*SP110c* mRNA and a significant increase in b*SP110a* and b*SP110b* mRNA (Figure 6A). These results indicate that SRSF1 is involved in human *SP110* splicing and affects the production of b*SP110c*. We mutated the ESE in the pre-SAND exon sequence of the human *SP110* gene in b*SP110*-Rhuman and co-expressed it with SRSF1, and the results showed that mutation of the ESE down-regulated b*SP110c* (Figure 6B). RNA immunoprecipitation (RIP) results showed that SRSF1 specifically bound to this ESE (Figure 6C). We used the same method to determine the effect of splicing factor SRSF5 on the variable splicing of b*SP110*. The results showed that co-overexpression of SRSF5 and bSP110-Rhorse effectively promoted the production of b*SP110b* mRNA and reduced the mRNA levels of b*SP110a* and b*SP110c*. We then co-expressed anti-SRSF5 siRNA and the b*SP110*-Rhorse vector, the results were opposite to those of overexpression of SRSF1, with a significant increase in b*SP110a* and b*SP110c* mRNA, and a significant decrease in b*SP110b* mRNA (Figure 6D).

## Discussion

Bovine tuberculosis (bTB) is classified by the World Organization for Animal Health as a notifiable animal disease [4, 5]. Although developed countries like the United Kingdom have reduced bTB incidence through long-term implementation of test-and-cull policies, the economic losses from culling may account for 30–50% of the disparity between the market value and slaughter value of dairy or beef cattle [25, 26]. In developing countries, the high prevalence of bTB, compounded by prohibitive control costs, poses significant threats to both animal-derived food safety and public health [27–29]. In addition, the control of bTB is made more difficult by the widespread presence of wildlife hosts, the lack of effective vaccines, and the evolution of MTB drug resistance [30–33]. Studies have shown that dairy cattle are highly susceptible to bTB and that this susceptibility is related to genetic traits [8–11]; therefore, identifying genetic factors of dairy cattle associated with bTB susceptibility and using gene editing to develop bTB-resistant cattle can be a cost-effective strategy for the control of bTB [34–37]. The focus of this research study was to investigate the genetic basis of the susceptibility of dairy cattle to bTB from the perspective of interspecies variation, and to identify potential gene-editing targets for breeding bTB-resistant cattle.

The *SP110* gene has been proven to be associated with host susceptibility to TB. *SP110* is a member of the speckled protein (SP) family, which contains three nuclear body components: *SP100*, *SP110* and *SP140* [38, 39]. SP family proteins are characterized by the presence of CARD, NLS, SAND, PHD and BRD domains [40], and the SAND domain is the core functional domain of *SP110* that exerts the anti-TB function [16, 41, 42]. The *SP110* gene shows complex alternative splicing [39], and the different splice variants have functional differences [21, 43, 44], affecting resistance to TB infection. Studies have indicated that *SP110* gene structure differs among species [20], and there is evidence that the *SP110* gene might have selectively evolved in ruminants [45, 46]. However, there has been little research on the bovine *SP110* gene, with only a few reports demonstrating that b*SP110* gene polymorphisms were associated with MTB susceptibility [47–49]. Our study is the first to examine the splice variants of the bovine *SP110* gene. We found that the b*SP110* gene can produce three splice variants with sequential deletions in C-terminal functional domains. The most abundant bovine splice variant was b*SP110c*, which lacks the core TB resistance domain SAND; *SP110c* has not been reported in other species.

**Figure 7:**
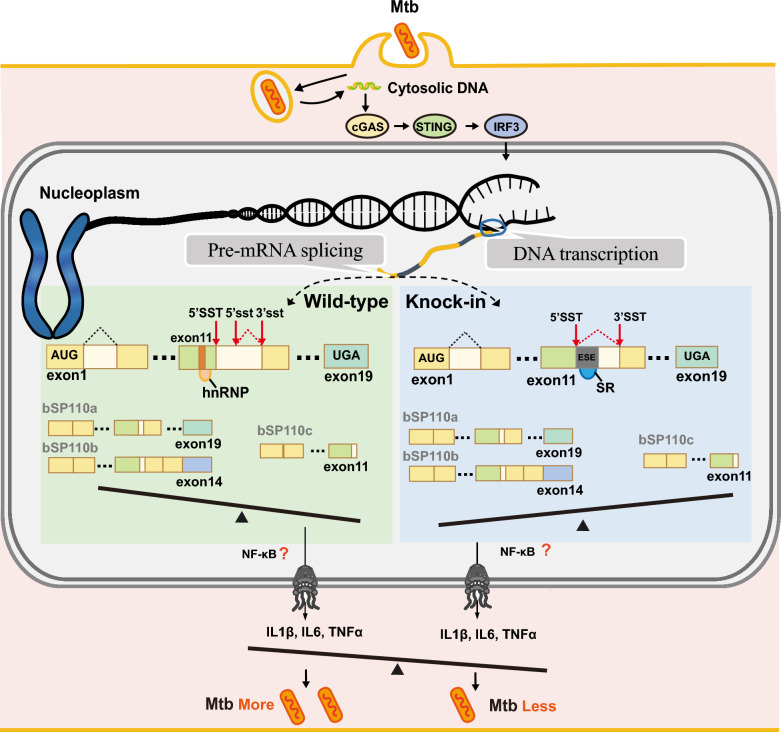
**Diagram of b*SP110* splicing, illustrating how cloning the human or horse pre-SAND exon into the b*SP110*** **gene can alter variable splicing and enhance the anti-bTB capability of macrophages.**

To investigate the generation of b*SP110c*, we first analyzed the b*SP110* gene structure. We found that intron 11 of b*SP110* contained not only the canonical 5′ splice site (5′ SST) but also a cryptic 5′ splice site (5′ sst). Competition between these two splice sites can lead to aberrant splicing, generating b*SP110c* when the spliceosome recognizes 5′ sst. Our study also revealed that hnRNPs regulate b*SP110c* splicing, suggesting the presence of an exon splicing silencer (ESS) in exon 11 of b*SP110* that binds hnRNPs leading to exon skipping and b*SP110c* formation. We also compared *SP110* gene structures across species, and discovered that ruminants lack the pre-SAND exon. Replacing the corresponding region of the b*SP110* gene with the pre-SAND exon sequences from humans or horses regulated alternative splicing, leading to a decrease in b*SP110c* and an increase in b*SP110a* and b*SP110b* expression. The results showed that both human and equine pre-SAND exons contained exon splicing enhancers (ESEs) capable of binding SR proteins. This ESE-SR protein interaction modulated alternative splicing of b*SP110* (Figure 7).

Although this research provides valuable insights, there are some limitations. We demonstrated that insertion of the pre-SAND exon can reduce the production of b*SP110c* and increase the production of b*SP110a* and b*SP110b*, but the effect of the pre-SAND exon on the resistance of macrophages to MTB infection requires further investigation with animal models, and results must be interpreted with caution. We observed that H37Ra, which is deficient in the ESX-1 system, can also significantly activate *SP110* gene expression. We hypothesize that this may be associated with mitochondrial stress [41], but this needs to be experimentally tested.

Altogether, these findings increase our understanding of the genetic factors involved in the susceptibility of dairy cattle to *M. bovis* infection. Our study provides a gene-editing site for the potential breeding of bTB-resistant dairy cattle.

## Supplementary Information

**Additional file 1 Primer sequences of b*SP110*** **gene PCR amplification**. The primers used for constructing the b*SP110* minigene eukaryotic expression vector.

**Additional file 2 Primer sequences of human and horse** ***SP110*** gene PCR amplification. The primers used to obtain the human or equine pre-SAND sequence.

**Additional file 3 Results of ESE analysis of human and horse** ***SP110*** **pre-SAND exon**. The results of the analysis performed using ESE Finder software, revealing ESE motifs within the human and equine *SP110* pre-SAND exon that can bind to SR proteins.

## Data Availability

All the data generated or analyzed in this study are included in this paper.
