# Supplementary material for: Species-specific alternative splicing of SP110 drives tuberculosis susceptibility in cattle
Source: Vet Res. 2025 Dec 12;57:10. doi: 10.1186/s13567-025-01644-3 (PMC12809954; doi:10.1186/s13567-025-01644-3)
Supplement: Supplementary file 1 — Additional file 1 Primer sequences of bSP110 gene PCR amplification. The primers used for constructing the bSP110 minigene eukaryotic expression vector. [file 13567_2025_1644_MOESM1_ESM.docx]

**Additional file 1：Primer sequences of bSP110 gene PCR amplification**

| Primer Name | Primer Sequence（5’-3’） |
| --- | --- |
| bSP110-EcoRI-F | CCgaattcGGATGACCAGGGCCTTGGAAAAGGCTC |
| bSP110a-XhoI-R | CGctcgagTCAAGGATGAGCCTGGAAG |
| bSP110b-XhoI-R | CGctcgagTCACCTGCTATTTACCTCTG |
| bSP110c-XhoI-R | CGctcgagTCACTTTAGATCTACATCAC |
| bSP110-exon1-F | CTACAAGGACGACGATGACaagcttATGACCAGGGCCTTGGAAAAGGCTC |
| bSP110- exon11-R | AGTAATCACTTTAGATCTACATCACCTGTGCGGGCTCCATGAGAGACC |
| bSP110-intron11-F5 | GGTCTCTCATGGAGCCCGCACAGGTGATGTAGATCTAAAGTGATTACT |
| bSP110-intron11-R5 | GAAAAGAAAACCCCCAAGAGTTCTAGACTAGCGGCCGGGTTTCGAGGAAAACAC |
| bSP110- intron11-F3 | GTGTTTTCCTCGAAACCCGGCCGCTAGTCTAGAACTCTTGGGGGTTTTCTTTTC |
| bSP110- intron14-R5 | GGAGGACAGAGGAGCGGCTAGCTCCTGACCTTAAACCCTTAG |
| bSP110- intron14-F3 | CTAAGGGTTTAAGGTCAGGAGCTAGCCGCTCCTCTGTCCTCC |
| bSP110- intron14-R3 | CACATTCCTTGGAGTTTTCCTGCTAGAAGGGGATTGGTACCTG |
| bSP110- exon15-F | CAGGTACCAATCCCCTTCTAGCAGGAAAACTCCAAGGAATGTG |
| bSP110- 3UTR-R | GTATCTTATCATGTCTGGATCCCCGCGGCCGCGAATGTGAACGGTGGTGAGG |
