# Supplementary material for: Species-specific alternative splicing of SP110 drives tuberculosis susceptibility in cattle
Source: Vet Res. 2025 Dec 12;57:10. doi: 10.1186/s13567-025-01644-3 (PMC12809954; doi:10.1186/s13567-025-01644-3)
Supplement: Supplementary file 2 — Additional file 2 Primer sequences of human and horse SP110 gene PCR amplification. The primers used to obtain the human or equine pre-SAND sequence. [file 13567_2025_1644_MOESM2_ESM.docx]

**Additional file 2：Primer sequences of human and horse SP110 gene PCR amplification**

| Primer Name | Primer Sequence（5’-3’） |
| --- | --- |
| human-SP110- exon12-F | GCCCGAATGCCTGCACAAAAGGAAAAGAAAAAGGAGAAAGATATC |
| human-SP110-intron12-R | GAATGTGTCCACCCCCGTCATCATTTTTGGGTCTTTGTATAG |
| horse-SP110-exon12-F | GCCCGAATGCCTGCACAAAAGGAAAAGATGAGAGAAAAATGTAGC |
| horse-SP110-intron12-R | GAATGTGTCCACCCCCGTCGTCCACCCCCACCTCCACTCC |
