# Supplementary material for: Species-specific alternative splicing of SP110 drives tuberculosis susceptibility in cattle
Source: Vet Res. 2025 Dec 12;57:10. doi: 10.1186/s13567-025-01644-3 (PMC12809954; doi:10.1186/s13567-025-01644-3)
Supplement: Supplementary file 3 — Additional file 3 Results of ESE analysis of human and horse SP110 pre-SAND exon. The results of the analysis performed using ESE Finder software, revealing ESE motifs within the human and equine SP110 pre-SAND exon that can bind to SR proteins. [file 13567_2025_1644_MOESM3_ESM.docx]

**Additional file 3：Results of ESE analysis of human and horse SP110 pre-SAND exon**

| SR Protein | ESE Number | Score |
| --- | --- | --- |
| human-SRSF1(SF2/ASF) | 7 | 2.41369 |
| human-SRSF1 (IgM-BRCA1) | 8 | 3.00752 |
| human-SRSF2(SC35) | 9 | 3.50868 |
| human-SRSF5(SRp40) | 15 | 2.58334 |
| human-SRSF6(SRp55) | 8 | 2.04413 |
| horse-SRSF1(SF2/ASF) | 9 | 1.58370 |
| horse-SRSF1 (IgM-BRCA1) | 7 | 1.70694 |
| horse-SRSF2(SC35) | 9 | 3.85856 |
| horse-SRSF5(SRp40) | 11 | 3.88775 |
| horse-SRSF6(SRp55) | 6 | 3.16165 |
